# Green Ultrasound-Assisted Synthesis of Rare-Earth-Based MOFs

**DOI:** 10.3390/molecules28166088

**Published:** 2023-08-16

**Authors:** Francesca Lo Presti, Anna L. Pellegrino, Nancy Consoli, Graziella Malandrino

**Affiliations:** Dipartimento di Scienze Chimiche, Università di Catania, and INSTM UdR Catania, Viale A. Doria 6, I-95125 Catania, Italy; francesca.lopresti@phd.unict.it (F.L.P.); annalucia.pellegrino@unict.it (A.L.P.);

**Keywords:** trimesic acid, yttrium-based MOF, morphological control, thermal stability, ultrasound, X-ray diffraction

## Abstract

Rare-earth (RE)-based metal organic frameworks (MOFs) are quickly gaining popularity as flexible functional materials in a variety of technological fields. These MOFs are useful for more than just conventional uses like gas sensors and catalyst materials; in fact, they also show significant promise in emerging technologies including photovoltaics, optical, and biomedical applications. Using yttrium and europium as ionic host centres and dopants, respectively, and 1,3,5-benzenetricarboxylic acid (H_3_-BTC) as an organic linker, we describe a simple and green approach for the fabrication of RE-MOFs. Specifically, Y-BTCs and Eu-doped Y-BTCs MOFs have been synthesised in a single step using an eco-friendly method that makes use of ultrasound technology. To establish a correlation between the morphological and structural properties and reaction conditions, a range of distinct reaction periods has been employed for the synthetic processes. Detailed analyses of the synthesised samples through powder X-ray diffraction (PXRD), field emission scanning electron microscopy (FE-SEM), and Fourier-transform infrared spectroscopy (FT-IR) have confirmed the phase formation. Furthermore, thermal analyses such as thermogravimetric analysis (TGA) have been employed to evaluate the thermal stability and structural modifications of the Y-BTC and Eu-doped Y-BTC samples. Finally, the luminescent properties of the synthesised samples doped with Eu^3+^ have been assessed, providing an evaluation of their characteristics. As a proof of concept, an Eu-doped Y-BTC sample has been applied for the sensing of nitrobenzene as a molecule test of nitro derivatives.

## 1. Introduction

The scientific community has recently devoted a lot of interest to the study of metal-organic frameworks (MOFs), a unique class of materials that exhibit a number of fascinating properties. The ability to modify these features by appropriately altering the MOFs’ structure, which can be changed by altering the metal centres, the organic linkers, and the synthesis’ operational parameters, including temperature, process length, etc., is the primary reason for this interest [1]. A subclass of MOFs called luminescent MOFs has lately attracted a lot of attention because of their prospective applications in areas including sensing [2,3], lighting technologies [4], selective separation [5], and biological fields [6,7,8,9,10].

The primary luminescent emission mechanisms discovered for these compounds can be categorised into groups like organic linker-based emission, such as ligand-to-metal charge transfer (LMCT) or metal-to-ligand charge transfer (MLCT), metal-based emission, and antenna effect [11]. Specifically, the type of metal utilised determines most of the luminescent characteristics of produced MOFs; these substances emit light at certain wavelengths via a mechanism of metal atomic relaxation. For instance, rare-earth-based MOFs (RE-MOFs) exhibit bright luminescence as framework ions; nevertheless, the strength of their luminescence can be boosted via the antenna effect [12,13]. To date, many RE-MOFs have been made using various organic linkers [14,15,16,17]. In particular, due to the presence of oxygen atoms in carboxyl groups, organic linkers such as 1,3,5-benzenetricarboxylic acid (H_3_-BTC) or 1,4-benzenedicarboxylic acid (H_2_-BDC) are able to coordinate metal ions to form highly stable three-dimensional networks [18,19]. Regarding the central metal, Y^3+^-based compounds are commonly used as matrices for traditional luminescent ions, such as Eu^3+^, Tb^3+^, and Tm^3+^ [20,21,22,23].

In regard to applications, RE-MOFs are now being developed as the most promising luminescent sensing materials for the detection of explosives [24], antibiotics [25], hazardous compounds and ions [26,27,28], heavy metals [29,30], and more. They have also been developed as catalysts [31], in thermometry [32,33], and in biological applications [34,35,36,37,38]. Additionally, the combination of RE-MOFs with polymeric structures could produce brand-new composite materials that merge the exceptional properties of MOFs with the features of the polymer supports [39,40,41,42].

Most RE-MOF synthetic approaches employed so far depend on solvothermal or hydrothermal techniques, which require high pressure, potentially hazardous organic solvents, and extremely lengthy reaction times [43,44,45,46]. There has not been too much effort, up to now, to change to a greener synthetic solution approach. For instance, Medina-Velazquez et al. [47] have developed Eu-BTC MOF nanopowders of high purity that are insoluble in water at room temperature in a basic medium. Zheng et al. [48] proposed the successful production of a variety of nano MOFs by chelating benzene tricarboxylic acid with various lanthanide ions on a wide scale by a one-step precipitation process in solution phase under ambient conditions. Recently, we performed a comprehensive study in which various reaction times and temperatures were tested in order to link the morphological and structural characteristics to the synthetic conditions [49]. This study has allowed us to better understand the mechanisms that would enable morphological control. Thus, while the morphology of the Y-BTC systems changes at higher synthetic temperatures with smaller and less homogenous crystals, the reaction time appears to favour a control in the development with an enhancement in morphological uniformity, both in terms of shape and size [49].

In the attempt to speed up the reaction process, reduce the reaction time, and switch to a greener method, several approaches have been developed, such as ultrasound (US) [50,51], microwave [52], mechanochemical synthesis [53,54], and electrodeposition [55].

In particular, the ultrasound method may increase the reactivity of the reagents, accelerate the crystallisation process, and produce homogeneous crystal particles with precise sizes [56]. In contrast, the reaction temperature and time, the solvents, and the process conditions have a strict influence on the grain size of the particles formed using techniques like hydrothermal or solvothermal synthesis.

Very few studies in the literature propose this convenient and fast synthetic method for the production of rare-earth-based MOFs [57,58,59]. More space is given to MOFs based on transition metals, such as Cu-BTC or even Zn-BTC, which are often used as catalysts [60,61].

In this study, we propose a green, facile, and rapid synthetic strategy to produce Y-BTC and Eu-doped Y-BTC MOFs using ultrasound (US) as a promising synthetic method. Specifically, the Y-BTC and Eu-doped Y-BTC MOFs have been synthesised under ultrasound at room temperature with reaction durations of 15, 30, 60, and 90 min. For the various Y-BTC and Eu-doped Y-BTCs samples, structural, thermal, and morphological characterisations have been carried out. Powder X-ray diffraction (PXRD), Fourier-transform infrared spectroscopy (FT-IR), thermogravimetric analyses (TGA), and differential scanning calorimetry (DSC) have all been used to evaluate the materials. Field emission scanning electron microscopy (FE-SEM) has been used to examine the morphology of powders that were directly deposited on aluminium stubs and sputtered with gold. This investigation provides a correlation between structure/morphology and synthetic process parameters. For the Eu-doped Y-BTC, preliminary luminescence characterisations and sensing of nitro derivatives have been carried out.

## 2. Results

### 2.1. MOF Synthesis

Using commonly accessible chemicals, water and ethanol as solvents, and mild pressure and temperature conditions, Y-MOFs have been synthesised through a straightforward ultrasound-assisted method. In several fields of chemical research, US-assisted syntheses have received a lot of interest. The possibility of carrying out chemical reactions at ambient temperature and pressure is one of the numerous advantages of ultrasonic technology. This ability is primarily attributed to the process of acoustic cavitation, which is the generation, growth, and collapse of bubbles, resulting in extraordinarily high local pressures and temperatures [62]. This approach has proven to be a successful means for synthesising MOFs at low reaction times, low reaction temperatures, and atmospheric pressure [57,58,59]. The syntheses have been carried out in water/ethanol solutions using 1,3,5-benzenetricarboxylic acid (H_3_-BTC) as the organic binder, commonly known as trimesic acid, and Y(CH_3_COO)_3_•xH_2_O and Eu(CH_3_COO)_3_•xH_2_O as precursors of Y^3+^ and Eu^3+^, the host metal centre and ionic dopant, respectively. Thus, two different types of systems have been synthesised: (i) pure Y-BTC MOFs with just yttrium as the metal centre, called Y-BTC; and (ii) Eu-doped Y-BTCs structures, named Y-BTC:Eu (10%). An Eu amount of 10% has been chosen to have enough luminescence signal for sensing applications, considering that usually pure Eu-MOFs are used for this purpose.

**Scheme 1:**
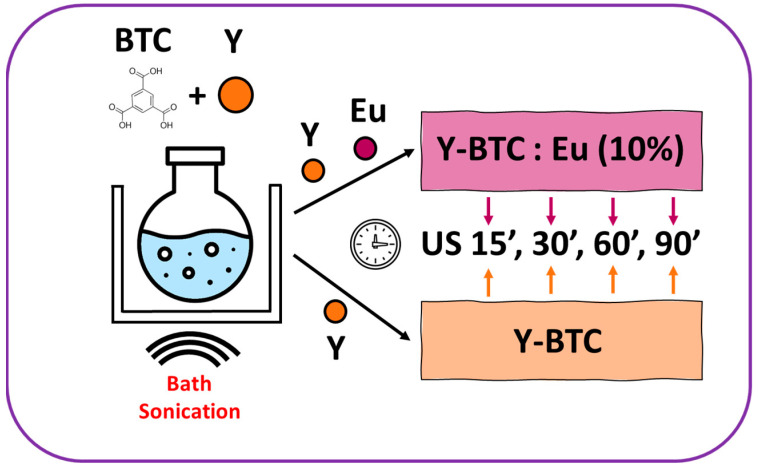
Scheme of the various syntheses of Y-BTC and Y-BTC: Eu (10%) under ultrasound and at different reaction times (15–90 min).

The syntheses were conducted under ultrasound at room temperature and atmospheric pressure for various reaction times according to the following reactions for Y-BTC (Equation (1)) and for Y-BTC:Eu (Equation (2)) and as reported in Scheme 1:

(1) $$Y({\mathrm{CH}}_{3}\mathrm{COO}{)}_{3}•{\mathrm{xH}}_{2}O+H_{3}-\mathrm{BTC}\;\overset{H_{2}O/EtOH}{\to }[Y(\mathrm{BTC})\cdot 6H_{2}O]+3{\mathrm{CH}}_{3}\mathrm{COOH}$$

(2) $$0.9\;Y({\mathrm{CH}}_{3}\mathrm{COO}{)}_{3}•{\mathrm{xH}}_{2}O+0.1\;\mathrm{Eu}({\mathrm{CH}}_{3}\mathrm{COO}{)}_{3}•{\mathrm{xH}}_{2}O+H_{3}-\mathrm{BTC}\;\overset{H_{2}O/EtOH}{\to }\;[Y_{0.9}{\mathrm{Eu}}_{0.1}(\mathrm{BTC})\cdot 6H_{2}O]+3{\mathrm{CH}}_{3}\mathrm{COOH}$$

### 2.2. Structural and Morphological Characterisation

**Figure 1:**
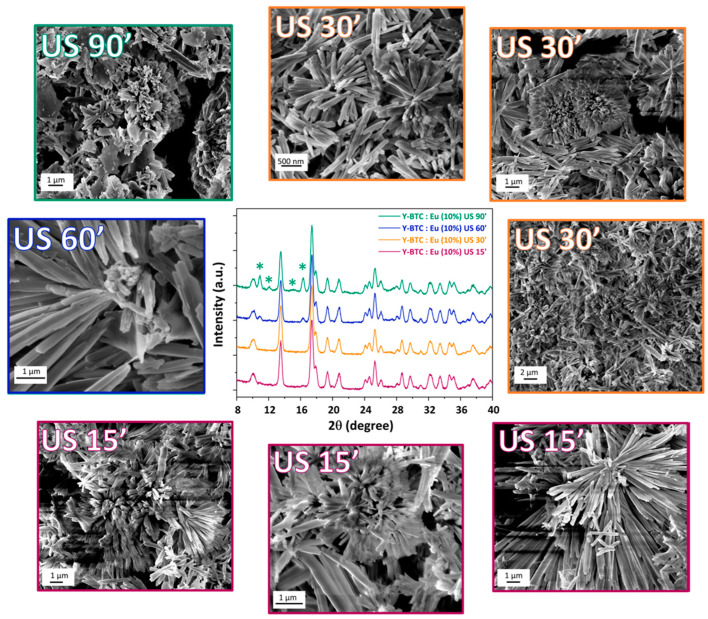
XRD patterns and FE-SEM images of Y-BTC:Eu (10%) samples produced under ultrasound with different reaction times: 15, 30, 60, and 90 min. * indicates peaks of the minority phase due to the novel crystal structure [Y(BTC)]_3_H_2_O [49].

For each synthesis of Y-MOFs and Y,Eu-MOFs, structural characterisations have been carried out concurrently through XRD and FE-SEM in order to ascertain if potential alterations or MOFs morphology changes are closely associated with their crystal structure. An overview of SEM images and XRD patterns for MOFs of Y-BTC:Eu (10%) made by ultrasonic-assisted synthesis is shown in Figure 1. The PXRD patterns of all Y-BTC:Eu (10%) syntheses are comparable. Specifically, for Y-BTC: Eu (10%) synthesised under ultrasound conditions for 15 (sample US 15′, reddish line) and 30 (sample US 30′, orange line) minutes, the crystal structures are consistent with the one described in ref. [63], which is coherent with the crystalline phase Y(BTC)(H_2_O)_6_. Figure S1 reports a comparison of the Y-BTC: Eu (10%) US 15′ with the Y(BTC)(H_2_O)_6_ (GOCYAY) structure [63]. This result finds a counterpart in the product observed in the green synthesis of Y-BTC MOFs at room temperature and atmospheric pressure in a water/ethanol solution [49]. Nonetheless, as the reaction time increases, peaks suggest the presence of a second phase that starts to develop alongside the main crystalline phase, e.g., see the Y-BTC:Eu (10%) US 60’ (blue line) and US 90’ (green line). This minority phase may be connected to a novel crystal structure recently described, different from all the other known structures, which consists of Y^3+^/BTC^3−^ units and a very small amount of water, [Y(BTC)]_3_H_2_O. Such structure was produced by employing a synthesis under reflux that took place in a water/ethanol solution for 24 h at a temperature of 80 °C [49].

Time in ultrasonic synthesis also significantly affects morphology, paralleling structural changes. The FE-SEM images obtained for Y-BTC: Eu (10%) synthesised under US for 15 min (reddish line) indicate that the sample is extremely inhomogeneous, with rod-like structures in the order of several tens of microns, porous flower-like structures, needle-like crystals, and others in the shape of bundles of tubes. Thus, highly inhomogeneous structures of all sizes, from those that are nearly “macroscopic” to those with dimensions in the tens of nanometers and a high aspect ratio, are present as a result of the first formed product. A reduction in morphological and dimensional inhomogeneity may be seen by reaching 30 min (Y-BTC: Eu (10%) US 30’—orange line), in which the flower and stick shapes are maintained. A further improvement in morphological and dimensional homogeneity is shown in the sample synthesised for 60 min (Y-BTC: Eu (10%) US 60’—blue line), with elongated stick-shaped structures having lengths on the order of tens of microns. With these reaction times, smaller, flattened, sheet-like structures also start to emerge. After 90 min of ultrasound, the morphology finally seems more altered than one may expect: the huge sticks start to break, and the leaflet morphologies become more apparent and bigger (US 90′, green line).

This alteration in morphology is likely to be connected to the structural alterations of Y-BTC:Eu (10%); at high synthetic times (US 60′ and US 90′), the variation in the structure and, therefore, in the morphology, suggests that it may be linked to the synthetic process. The ultrasonic cavity procedure might produce free radicals that cause the material’s structure to change as a result of the high pressure and high temperature conditions that are locally present in the reaction environment. The frequency of ultrasound-induced collisions increases with time, accelerating the interaction between the substrate and the reactant and likely leading to structural and morphological changes.

Energy dispersive X-ray analysis has also been performed on all the Eu-doped Y-BTC samples to gather qualitative and quantitative data on the presence of europium in the structures. As a proof case, the EDX spectrum of the US 90′ sample is reported in Figure S2. The X-ray L lines of Eu and Y are observed in the range 5.8–6.5 keV and at 1.92 keV, respectively, in addition to the C K_α_ peak found at 0.28 keV and the O K_α_ peak at 0.524 keV. In particular, the quantitative analysis gives a percentage of Eu doping in the sample of 9.5 ± 0.5%.

**Figure 2:**
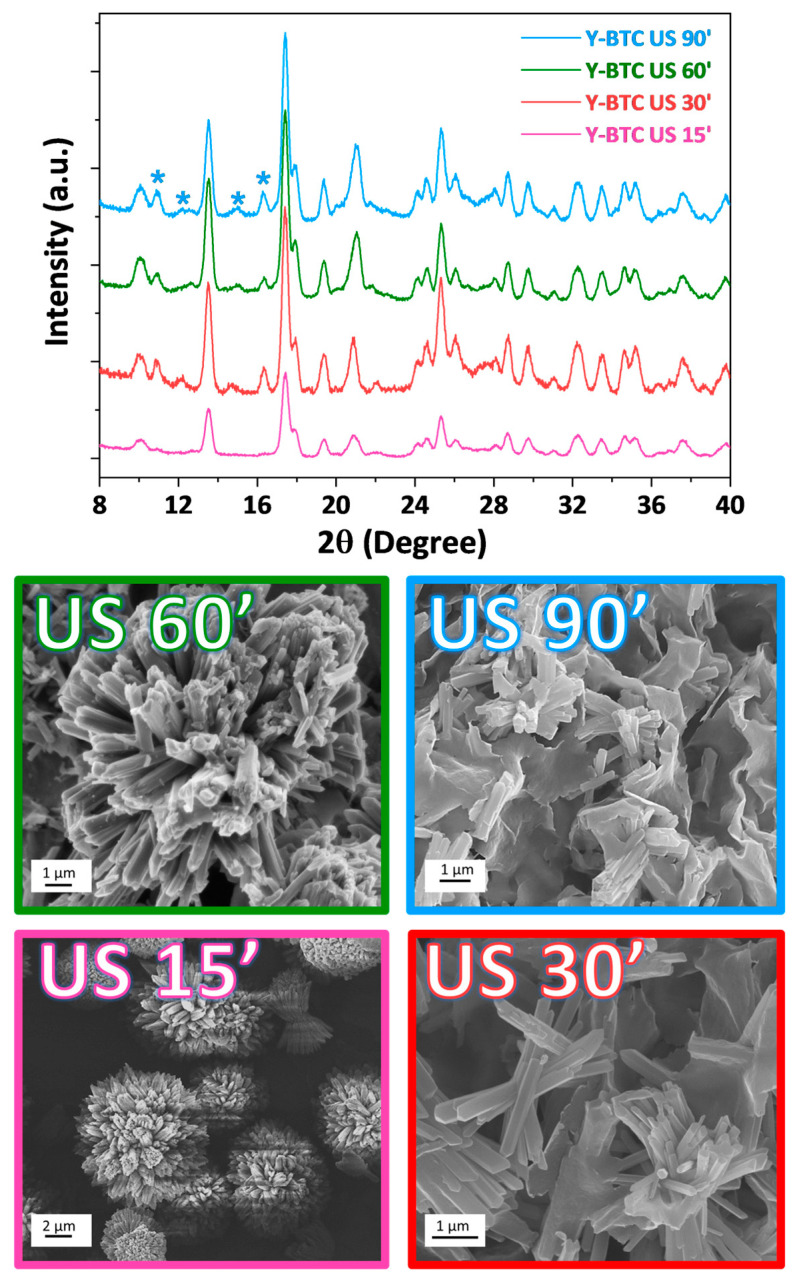
XRD patterns and FE-SEM images of Y-BTC samples synthetised under ultrasound for different reaction times: 15, 30, 60, and 90 min. * indicates peaks of a minority phase due to the novel crystal structure [Y(BTC)]_3_H_2_O [49].

A similar situation in terms of structural and morphological characteristics is found for the undoped Y-BTC MOF synthesised by ultrasound for 15, 30, 60, and 90 min. The XRD patterns of Y-BTC samples synthesised at various times are displayed in Figure 2. For the undoped samples, it is also evident that the Y-BTC US 15′ sample produced a pattern analogous to the structure of ref. [63]. The two samples of Y-BTC and Y-BTC: Eu (10%) synthesised for 15’ appear to be isostructural with the GOCYAY structure [63], in which yttrium is nona-coordinated by six oxygen atoms from water molecules as well as three oxygen atoms from three carboxylate groups to form a tricapped trigonal prism (Figure S3). A comparison of the three patterns of Y-BTC US 15′, Y-BTC: Eu US 15′, and GOCYAY is reported in Figure S1. On increasing reaction time, peaks corresponding to a second crystalline phase appear for samples Y-BTC US 30’, 60’, and 90’, analogously to what is observed for the Eu-doped Y-BTC MOFs.

The morphology of the US samples of Y-BTC similarly displays the same characteristics as those of the doped samples. In fact, the Y-BTC US 15’ MOF (pink line) mostly depicts heterogeneous structures, with a tendency for stick-like growths of various sizes and shapes to eventually take on the appearance of flowers. The morphological inhomogeneity starts to reduce as the reaction time increases (US 30’—blue line), and the sticks that have developed have thicker and longer lengths. Furthermore, sheet-like forms start to emerge. The MOF sticks keep expanding in size and form for the Y-BTC US 60’ sample (green line), reaching lengths in the tens of microns. The stick structures finally seemed fractured after reaction times of 90 min (blue line), so the sticks changed into bigger sheet-like shapes.

**Figure 3:**
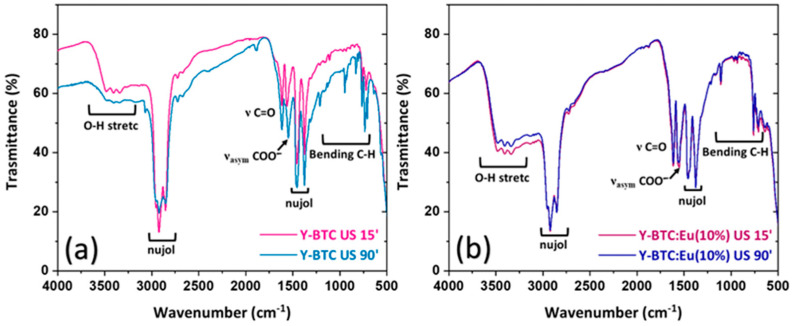
FT-IR spectra of (**a**) Y-BTC US 15′ and Y-BTC US 90′; (**b**) Y-BTC: Eu (10%) US 15′ and Y-BTC: Eu (10%) US 90′.

The FT-IR spectra of the Y-BTC and Y-BTC: Eu (10%) samples synthesised for 15 and 90 min provide additional support to the observations made by PXRD patterns. The FT-IR spectra of Y-BTC US 15’ and Y-BTC US 90’ are shown in Figure 3a. According to the degree and type of hydration in the two systems, these spectra demonstrate the existence of multiple peaks in the 3200–3500 cm^−1^ region, more pronounced for the US 15’ sample, associated with the -OH stretching. The nujol C–H stretching causes the signal at 2900 cm^−1^ to appear. Peaks at 1617 and 1560 cm^−1^ are related to the vibrations of the BTC ligand, and in particular to the stretching of the C=O and the asymmetric stretching of the COO^-^, respectively. The symmetric stretching of the COO^-^, usually observed around 1400 cm^−1^, is buried under the peaks due to the nujol oil at 1458 and 1375 cm^−1^.

Additional peaks seen in the fingerprint region (1000–700 cm^−1^) can be accounted for by the vibrational modes of the benzene rings and, in particular, the C-H bending vibrations. As the PXRD patterns revealed a similar crystalline phase, it is expected that the FT-IR spectra of the Y-BTC:Eu (10%) samples are equivalent to those of the parent undoped ones. Figure 3b shows the FT-IR spectra of the Eu-doped Y-BTCs synthesised for 15 and 90 min. A sequence of peaks at 3323, 3406, and 3485 cm^−1^, are present in the area of -OH stretching, suggesting the presence of water molecules coordinated to the metal ion (either Y or Eu). Peaks at 1614 and 1562 cm^−1^ represent the C=O stretching vibration and the COO^-^ asymmetric stretching, respectively, associated with the BTC ligand inside the MOF structure. Peaks related to the C–H bending vibrations are seen in the range of 1000–700 cm^−1^.

### 2.3. Thermal Properties

**Figure 4:**
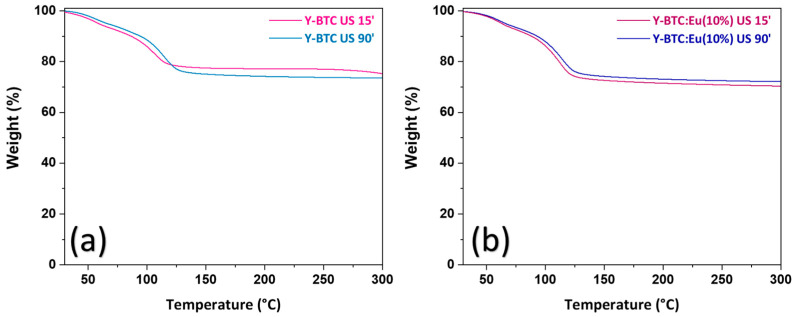
TGA curves of (**a**) Y-BTC US 15′ and Y-BTC US 90′; (**b**) Y-BTC: Eu (10%) US 15′ and Y-BTC: Eu (10%) US 90′.

Thermogravimetric measurements have been used to investigate the Y-BTC MOF’s thermal stability and confirm the presence of water molecules. As a result, the thermal characterisation of these Y-BTC samples is useful in identifying the potential presence and quantity of coordinated water molecules inside the MOF structure. The TGA curves of the Y-BTC US 15’ (pink line) and Y-BTC US 90’ are shown in Figure 4a (light blue line). For the Y-BTC US 15’, a first weight loss step of about 6.9% can be seen at 66 °C, which is probably the result of the loss of the first water molecule per unit formula Y(BTC)(H_2_O)_6_, while a second, larger weight loss step of 26.1% can be seen at 125 °C, which is the result of the loss of the additional five H_2_O molecules that coordinate the metal centre. The Y-BTC US 90’ exhibits a similar situation, showing three distinct weight loss steps of 4.4%, 8.4%, and 23.1% at temperatures of 62 °C, 86 °C, and 126 °C, respectively. These steps correspond to the loss of roughly 5.4 H_2_O molecules over one Y(BTC) unit.

The curves show similar thermal behaviour for the Y-BTC: Eu samples (Figure 4b). In particular, the graphs reveal two distinct weight losses at 72 °C and 120 °C. The first step often results in the loss of 1.5 molecules of water per unit formula, whereas the second step removes all 4 molecules of water that are still present, leaving behind a residue that is compatible with anhydrous Y-BTC.

The results of the thermal analysis are consistent with the infrared spectra, which show peaks due to numerous -OH stretching, and with the XRD patterns, which show for all the pure and Eu-doped Y-BTC samples a pattern corresponding to the Y(BTC)(H_2_O)_6_ structure.

### 2.4. Luminescence Characterisation

**Figure 5:**
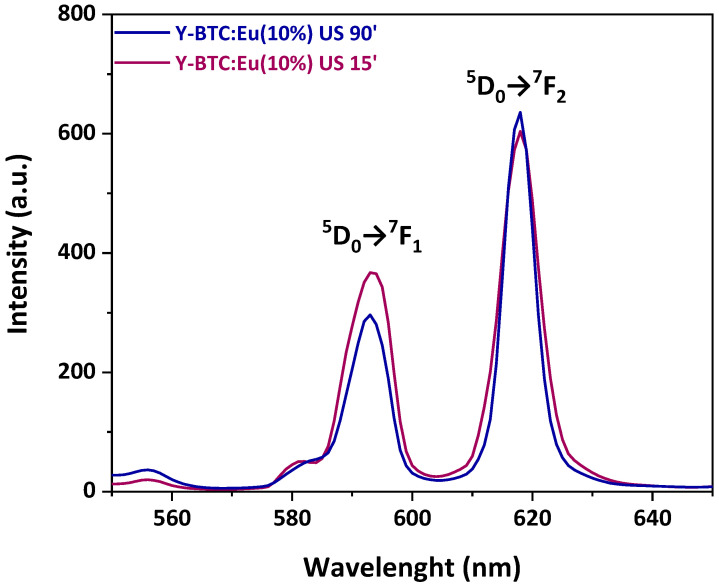
Luminescence spectra of Y-BTC US 15′ and Y-BTC US 90′, λ_exc._ = 265 nm.

Using dispersions of the Y-BTC: Eu (10%), US 15′, US 30′, US 60′, and US 90′ samples in aqueous media, photoluminescence measurements have been used to perform preliminary luminescent characterisation (see Figure 5). A slight effect of the MOF’s crystal structure is visible in the luminescence intensity of the various samples. Thus, no difference is found for the samples produced at 15, 30, and 60 min, where just one, or at least a dominant, crystalline phase is visible. In any case, the presence of a second crystalline phase in the sample produced at 90 min does not significantly affect the luminescence behaviour of the sample. At an excitation wavelength of 265 nm, all Eu-doped Y-MOF exhibit fluorescence emission. In Figure 5, the spectra of the Y-BTC: Eu (10%) US 15′ and Y-BTC: Eu (10%) US 90′ are reported. The strong peaks centred at 592 and 618 nm may be attributed to the ^5^D_0_ → ^7^F_1_ and ^5^D_0_ → ^7^F_2_ transitions, respectively, which represent two of the typical emissions of the Eu^3+^ ion. It is interesting to evaluate the asymmetry ratio *R*, i.e., the ratio between the integrated areas of the ^5^D_0_ → ^7^F_2_ and ^5^D_0_ → ^7^F_1_ electronic transitions, which can give a hint on the symmetry of the Eu^3+^ ion. In both cases, the R values of 1.43 and 1.65 for the Y-BTC: Eu (10%) US 15′ and Y-BTC: Eu (10%) US 90′, respectively, are indicative of a quite symmetric environment [64] for the Eu^3+^ ion, as expected for a tri-capped trigonal prismatic coordination, the coordination of europium when substituting yttrium in the Y(BTC)(H_2_O)_6_ structure (Figure S3).

The Y-BTC: Eu (10%) sample synthesised under ultrasound may hold promise as a potential luminescent material for different applications and other practical uses due to its strong emission, even for samples produced with very short reaction times.

### 2.5. Sensing of Nitro Derivatives

The Y-BTC: Eu (10%) samples synthesised under ultrasound may hold promise as potential systems for the detection of nitro derivatives.

Nitroaromatic chemicals are the principal components of explosives used in acts of terrorism and in improvised explosive devices. Thus, there is a critical need to develop reliable, user-friendly sensing materials for identifying nitroaromatic pollutants. As a proof of concept, the Y-BTC: Eu (10%) MOF 90′ sample, chosen because it is well crystallised and has a quite homogeneous morphology, has been applied as a fluorescent probe for sensing nitrobenzene (C_6_H_5_NO_2_ or NB), a test molecule for nitro derivatives.

**Figure 6:**
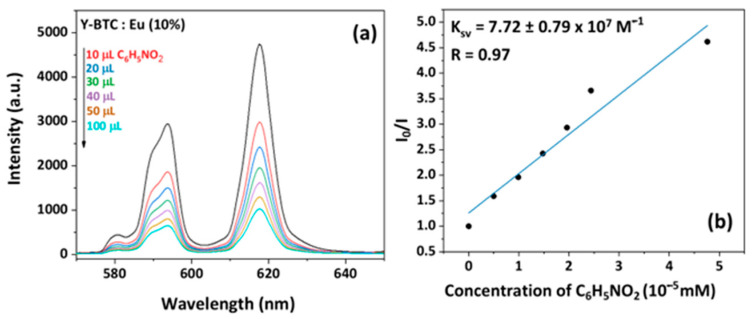
(**a**) Emission spectra and (**b**) K_sv_ curve of Y-BTC: Eu (10%) dispersed into aqueous solution (1 mg in 2 mL) in the presence of various concentrations of NB under excitation at 265 nm.

Aqueous solutions have been used to disperse the as-prepared Y-BTC:Eu MOF US 90’ (1 mg in 2 mL of H_2_O), and different quantities of C_6_H_5_NO_2_ (10^−6^ M solution in H_2_O) have been added to the suspension. To guarantee equal distribution and avoid sedimentation effects, the suspension has been sonicated for five minutes after each addition to produce suspensions suitable for luminescent measurements. The choice of a 5 min period of sonication allowed for the best possible interaction between the MOF and the identified species. Figure 6a illustrates a pronounced quenching effect on both the ^5^D_0_ → ^7^F_1_ and ^5^D_0_ → ^7^F_2_ peak intensities of the Eu^3+^ emission, which becomes evident even after the addition of just 10 μL of NB contaminant. To quantitatively assess this phenomenon, the Stern–Volmer (SV) equation I_0_/I = 1 + K_sv_[M] [65] has been employed. The I_0_ and I represent the intensity values of the most intense peaks (617.5 nm) of the Y-BTC: Eu spectrum in the absence and presence of analytes, respectively. K_sv_ is the quenching constant, and [M] denotes the absolute concentration of the analyte. The linear coefficients R, close to one, reveal strong linear correlations and a good fit for the Stern–Volmer equation model.

Furthermore, using the K_sv_ equation, the quenching impact of the aromatic component nitrobenzene is expected to be 7.72 ± 0.79 × 10^7^ M^−1^, indicating a very strong quenching effect on the Eu^3+^ luminescence. The detection mechanism behind this intriguing behaviour is attributed to the photo-induced intermolecular electron transfer between the MOF sensor and the analyte, as assessed through density functional theory (DFT) and time-dependent DFT [66,67].

The presently found K_sv_ value is higher than most of the previous works [68,69,70,71,72], proving the excellent sensitivity of the Eu-doped Y-BTC MOF to detect nitroaromatic compounds.

These findings demonstrate a promising outcome considering that presently only a 10% Eu-doped sample has been used, making the testing technique less expensive than using pure Eu-MOF structures.

## 3. Discussion

The ultrasound-assisted synthetic approach described in this study represents a green, quick, facile, and reproducible preparation method that can be used for the synthesis of Y-based MOFs in contrast to the existing synthetic methods reported in the literature, which frequently involve numerous steps, high temperatures, toxic solvents, and long reaction times. The US synthetic method has been demonstrated to be an effective approach for producing MOFs with fast crystallisation times, low reaction temperatures, and atmospheric pressure. This straightforward but effective synthetic method may be compared with that described in ref. [59], which describes the production of isostructural Ln(BTC) MOFs (Ln = Ce, Tb, and Y) with a tetragonal structure at room temperature through ultrasonic irradiation. In contrast to the previous study that focused on the minimum times for the full crystallisation of the Ln-MOF, the reaction conditions used in the present study have made it possible to conduct a more in-depth analysis of the crystallisation process and correlate the morphology and structure of the various samples to reaction conditions. The dopant’s contribution to both structural and luminescence properties of the Y-MOF structure could also be examined. For instance, it was shown that regardless of the dopant’s presence, the sample crystallinity tends to increase as the reaction time increases. The Y-MOF crystal structure of all the synthesised samples with different reaction times is comparable to that of ref. [63]. However, as reaction time increases, peaks that correspond to a second crystalline form start to develop. Such a new secondary phase consists of Y^3+^/BTC^3−^ units and very little water. From a morphological perspective, reaction time in ultrasonic synthesis promotes an improvement in shape distribution and a decrease in length that is more evident at 90 min of reaction. In fact, several types of morphologies, such as rod-like, flower-like, and needle-like structures, may be observed for Y-MOFs, both doped and undoped, prepared at short reaction durations, i.e., 15 and 30 min. The 60 min samples have a more uniform morphology, with stick-shaped structures on the order of tens of microns. The luminescent features of these MOFs, caused by the presence of Eu ions introduced as dopants into the Y-BTC structures, partly correlate with the synthesis time and, therefore, the crystallinity of the final samples. The luminescence behaviour of the Y-BTC: Eu (10%) US 15’ and 90’ is similar, indicating that the fluctuation in the MOF crystal structure does not significantly affect the emission of the Eu ion.

## 4. Materials and Methods

### 4.1. Materials

Yttrium(III) acetate hydrate and Europium(III) acetate hydrate were purchased from STREM Chemicals, and the 1,3,5-benzenetricarboxylic acid was purchased from Sigma-Aldrich. All the reagents were used without other purifications. Two different sets of syntheses have been performed. Scheme 1 provides a summary of the process.

### 4.2. Synthesis under Ultrasound

Y-BTC: Eu (10%). 50 mL of a water solution containing 3.6 mmol of Y(CH_3_COO)_3_•xH_2_O and 0.4 mmol of Eu(CH_3_COO)_3_•xH_2_O were added to a glass balloon with a grounded neck. After that, the solution was mixed with 50 mL of ethanol containing H_3_-BTC (4 mmol), and the system was exposed to ultrasound for various lengths of time: 15, 30, 60, and 90 min. The products were simply filtered out after the various reaction periods, and the Eu-doped Y-BTC precipitate was recovered, cleaned with water/ethanol, and allowed to dry in the open air.

Y-BTC. The undoped chemical products were obtained using a procedure similar to that used to create the related Eu-doped Y-BTC product, starting with 50 mL of a water solution containing 4 mmol of Y(CH_3_COO)_3_•xH_2_O and 50 mL of ethanol containing 4 mmol of H_3_-BTC acid. The durations of the ultrasonic treatments on the system were 15, 30, 60, and 90 min, respectively. The product was filtered before being rinsed with water and ethanol and dried in the air.

### 4.3. Characterisations

Fourier transform infrared (FT-IR) spectra on a JASCO FTIR 4600 LE spectrometer (Easton, MD, USA) were collected. A small amount of sample powder had been thoroughly pulverised in an agate mortar in nujol and placed between NaCl plates, which underwent FT-IR analysis. The Mettler Toledo TGA2 and STARe software were used to conduct thermogravimetric experiments. A 50 sccm pure nitrogen flow, atmospheric pressure, and a 5 °C/min heating rate were used in the dynamic thermal investigations. The weights of the samples were around 10 mg. With a Smartlab (Rigaku, Tokyo, Japan) diffractometer, XRD patterns were captured using a rotating Cu K_α_ anode operating at 45 kV and 200 mA. During the acquisition, a 0.02° increment step was used. The ZEISS SUPRA 55VP field-emission scanning electron microscope was used to carry out the morphological characterisation. Samples were attached to Al stubs using graphite double-sided adhesives before being sputtered with Au in order to characterise the morphology of the synthesised Y-MOF using the FE-SEM apparatus. Using energy dispersive X-ray (EDX) analysis by means of an INCA-Oxford windowless detector with a resolution of 127 eV, which was calculated as the full width half maximum (FWHM) of the Mn K_α_, the atomic composition of the films was determined. A JASCO FP-8300 spectrofluorimeter was used to capture photoluminescence spectra at room temperature with an excitation wavelength of 265 nm. In 2 mL of water, 2 mg of Y-BTC: Eu (10%) was dispersed for each experiment. The sensing test was carried out by adding different amounts of C_6_H_5_NO_2_ (10^−6^ M in H_2_O) to the Y-BTC: Eu (10%) dispersion (1 mg in 2 mL of H_2_O). Before starting additions, the Y-BTC: Eu dispersion was sonicated for half an hour to have a stable suspension. After each addition, the resulting mixtures were sonicated for 5 min to favour interaction and prepare them for luminescence measurements. The sequential C_6_H_5_NO_2_ detection tests used the same Y-BTC: Eu MOF powder.

## 5. Conclusions

In summary, this research study offers a simple, quick, and environmentally friendly method for creating several luminescent Eu-doped Y-BTC and undoped Y-BTC MOFs. Notably, the syntheses have applied ultrasonic technology under standard pressure and room temperature while just changing the reaction time. The extensive characterisations conducted in this work consistently demonstrate a repeatable process where the structural properties of the Y-BTCs and the synthetic process parameters are closely related. From a structural point of view, each sample synthesised using different reaction times displays a dominant phase linked to the crystalline Y(BTC)(H_2_O)_6_ MOF. However, as the reaction time increases, a secondary phase that shows the Y^3+^/BTC^3−^ units and a little quantity of water in the crystalline phase appears. Numerous morphologies, including porous flower-like formations, needle-shaped crystals, and tube-like structures, were seen in those synthesised with shorter reaction times (i.e., 15 and 30 min). However, as the reaction time increased, some of these structures began to sinter, giving rise to filamentous or tubular structures with higher aspect ratios. Eventually, the structures completely broke down after very lengthy reaction periods, like 90 min. Additionally, the 15 and 90 min samples have been shown to be highly stable, after losing the coordinated water molecules, up to 400 °C. The Eu-doped Y-BTC samples have comparable luminescent properties. This study represents a facile, general route to synthesise a variety of RE-doped MOFs, envisaging the possibility of tuning the specific properties in function of the desired sensing applications.

## Data Availability

Data are contained within the article or Supplementary Materials.

## Supplementary Materials

The following supporting information can be downloaded at: https://www.mdpi.com/article/10.3390/molecules28166088/s1, Figure S1: Comparison of the Y-BTC US 15′, Y-BTC: Eu US 15′ and GOCYAY-Y(BTC)(H_2_O)_6_ structures; Figure S2: EDX spectrum of the Y-BTC: Eu US 90′ sample; Figure S3: Coordination of the Y in the Y(BTC)(H2O)_6_ structure.
