# Supplementary material for: Green Ultrasound-Assisted Synthesis of Rare-Earth-Based MOFs
Source: Molecules. 2023 Aug 16;28(16):6088. doi: 10.3390/molecules28166088 (PMC10458194; doi:10.3390/molecules28166088)
Supplement: Supplementary file 1 [file molecules-28-06088-s001.zip › molecules-2531063-supplementary.pdf]

SUPPORTING INFORMATION

Green ultrasound assisted synthesis of rare-earth-based MOFs

Francesca Lo Presti, Anna Lucia Pellegrino, Nancy Consoli, Graziella Malandrino*

Dipartimento di Scienze Chimiche, Università di Catania, and INSTM UdR Catania, Viale A. Doria 6, I-95125 Catania, Italy.
*Correspondence: gmalandrino@unict.it

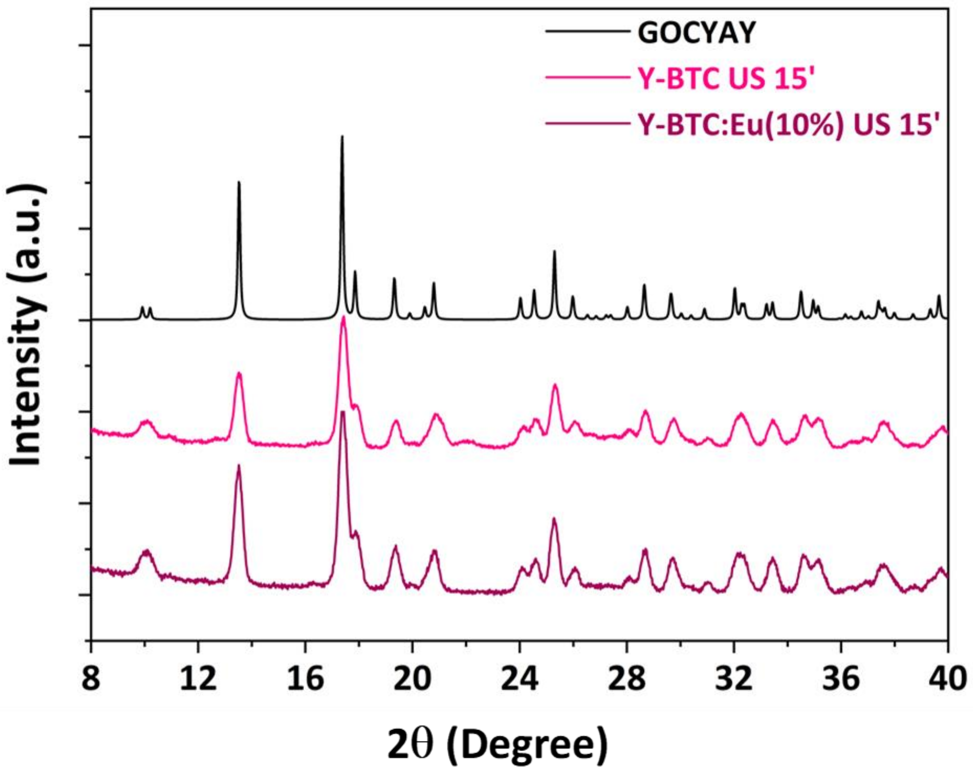

Figura S1. Comparison of the Y-BTC US 15’, Y-BTC: Eu US 15’and GOCYAY-Y(BTC)(H_2O)$_6$ structures.

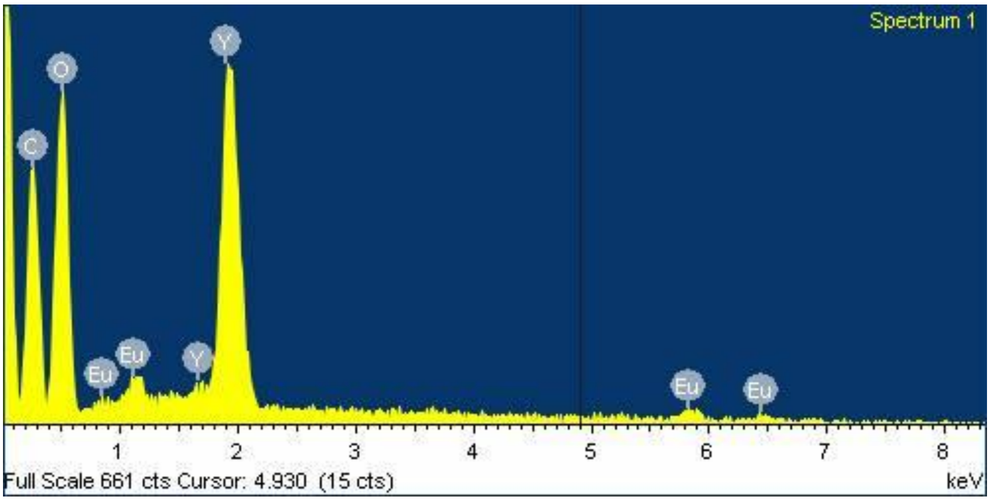

Figura S2. EDX spectrum of the Y-BTC: Eu US 90’ sample.

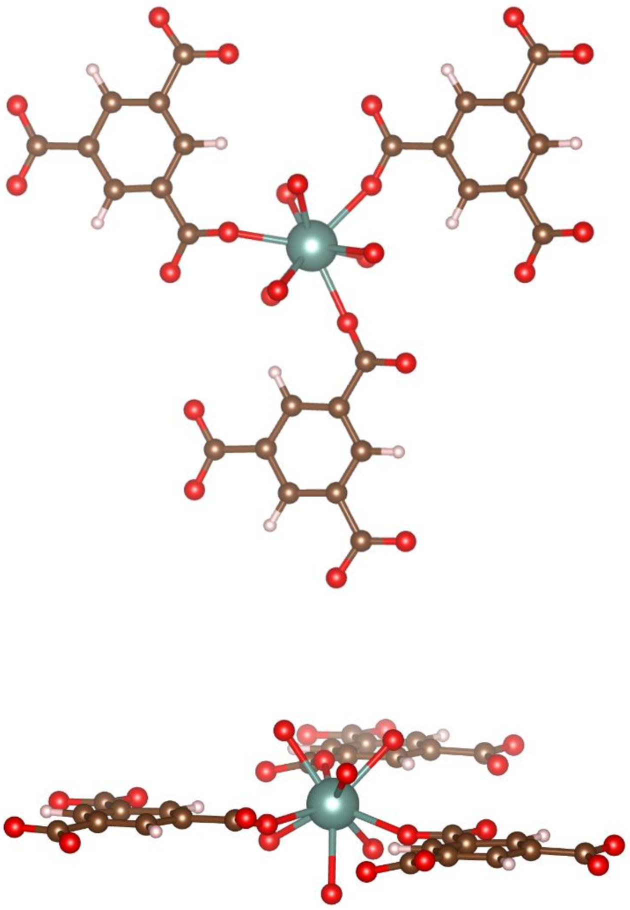

Figura S3. Coordination of the Y in the $Y(BTC)(H2O)_6$ structure obtained from the cif of GOCYAY structure [59] using the Vesta program.
